# Comparison of the effect of ultrasounic-harmonic scalpel and electrocautery in the treatment of axillary lymph nodes during radical surgery for breast cancer

**DOI:** 10.1186/s12957-024-03381-x

**Published:** 2024-04-11

**Authors:** Yujia Tian, Lifei Han, Xiao Ma, Rui Guo, Zhuoga GeSang, Yabo Zhai, Haolin Hu

**Affiliations:** 1 School of Medicine, Southeast University, Nanjing, 210009 China; 2 Department of General Surgery, Zhongda Hospital Affiliated to Southeast University, Nanjing, 210009 China; 3 Breast Disease Diagnosis and Treatment Center, Zhongda Hospital Affiliated to Southeast University, Nanjing, 210009 China

**Keywords:** Ultrasounic-harmonic scalpel, Electrocautery, Breast cancer, Modified radical mastectomy, Breast sparing mastectomy, Axillary lymph node treatment

## Abstract

**Objective:**

To compare the efficacy of ultrasounic-harmonic scalpel and electrocautery in the treatment of axillary lymph nodes during radical surgery for breast cancer.

**Methods:**

A prospective study was conducted in the Department of Breast Surgery, Zhongda Hospital Affiliated to Southeast University. A total of 128 patients with pathologically confirmed breast cancer who were treated by the same surgeon from July 2023 to November 2023 were included in the analysis. All breast operations were performed using electrocautery, and surgical instruments for axillary lymph nodes were divided into ultrasounic-harmonic scalpel group and electrocautery group using a random number table. According to the extent of lymph node surgery, it was divided into four groups: sentinel lymph node biopsy, lymph node at station I, lymph node at station I and II, and lymph node dissection at station I, II and III. Under the premise of controlling variables such as BMI, age and neoadjuvant chemotherapy, the effects of ultrasounic-harmonic scalpel and electrocautery in axillary surgery were compared.

**Results:**

Compared with the electrosurgical group, there were no significant differences in lymph node operation time, intraoperative blood loss, postoperative axillary drainage volume, axillary drainage tube indwelling time, postoperative pain score on the day after surgery, and the incidence of postoperative complications (*p*>0.05).

**Conclusion:**

There is no significant difference between ultrasounic-harmonic scalpel and electrocautery in axillary lymph node treatment for breast cancer patients, which can provide a basis for the selection of surgical energy instruments.

The number of new cases of breast cancer is increasing rapidly, and it has become the largest cancer around the world. Among female malignant tumors, the incidence of breast cancer was in the first place, which continues to increase [1–3]. Breast cancer treatment includes radiotherapy, chemotherapy, surgery, endocrine therapy and so on. Surgical treatment usually involves removal of the breast and axillary lymph nodes.

At present, high-frequency electrocautery is routinely used for surgical treatment. In addition, with the continuous development of medical instruments, ultrasounic-harmonic scalpel (UHS) is gradually widely used as a new surgical instrument. Axillary treatment for breast cancer (BC) patients has changed significantly over the past few years due to the declining status of multimodal approaches and axillary surgery as a staging procedure. This process of surgical downgrading began when anterior sentinel lymph node biopsy (SNB) replaced axillary lymph node dissection (ALND) as the standard treatment for patients with node-negative breast cancer [4]. With the publication of practice changing trials such as ACOSOG Z0011, IBCSG 23-01, and EORTC AMAROS [5–8], the gradual reduction in surgical interventions continues. ALND remains the standard treatment for clinically node-negative BC-positive patients who do not meet the eligibility criteria for these trials.

UHS is a safe and reliable surgical hemostatic device. UHS is mainly composed of main components such as host, handle, shears and foot pedal. There is a transducer inside the handle, which converts high-frequency electrical energy into ultrasonic mechanical vibration energy and transmits it to the shears for hemostatic cutting or blood clotting. The working temperature of UHS is 85°C, the thermal effect range is 1 ~ 2mm^2^, and the working frequency is 55.5kHz. UHS is equipped with a 10mm Laparosonic Coagulating Shears (LCS). The vibration amplitude of the shears is 50~80 μm.

The electrocautery uses high-frequency current generator to generate high-frequency current, which makes the local tissues of the body produce high temperature. The tissues of the body are separated, cut and solidified. The operating temperature of electrocautery is 150°C, the thermal effect range is 2.5 ~ 4mm^2^, and the working power output is set to 50 ~ 70W. The electrocautery is equipped with a 5mm electric hook.

The purpose of this study was to compare the efficacy of UHS and electrocautery in axillary lymph node treatment during radical breast cancer surgery, and to provide a basis for the selection of surgical energy instruments.

## Data and methods

### Research design

A total of 128 patients with breast cancer admitted to the Breast surgery Department of our hospital from July 2023 to November 2023 were included in the analysis. Inclusion criteria: ① From July 2023 to November 2023, they were treated by the same surgeon in the Breast Disease Center of Zhongda Hospital Affiliated to Southeast University, and pathologically confirmed to be breast cancer; ② Female patients aged 18-90 years; ③ Surgical procedures include: a. unilateral modified radical mastectomy (sentinel lymph node exploration, and/or axillary lymph node dissection); b. Unilateral breast sparing mastectomy (sentinel lymph node exploration, and/or axillary lymph node dissection). Exclusion criteria: ① bilateral breast cancer patients; ② Male breast cancer patients. Breast cancer staging relies on the TNM system designed jointly by the UICC (Union Internationale Against cancer) and the AJCC (American joint Committee on cancer). This study was reviewed and approved by the Medical Ethics Committee of Zhongda Hospital Affiliated to Southeast University (Approval number: 2023ZDSYLL231-P01). All enrolled patients had obtained written informed consent.

### Experimental methods

Under general anesthesia, the patients in both groups were taken to the horizontal position and treated according to standard modified radical mastectomy and breast sparing mastectomy. All breast operations were performed with electrocautery. The surgical instruments for axillary lymph nodes were divided into UHS group and electrocautery group using a random scale. According to the 2024 NCCN Breast Cancer Clinical Practice guidelines [9], the appropriate lymph node operation was selected based on the clinical stage,TNM stage and axillary lymph node metastasis. The UHS group was used to perform axillary lymph node operation with the UHS (Hou Ke Tianjin Medical Technology Co., LTD., Model: USE14). The small lymphatic vessels and blood vessels were directly severed and only the lateral thoracic vessels were ligated. During sentinel lymph node biopsy, 2mL 0.1% methylene blue injection (Jichuan Pharmaceutical Group Co., LTD., Sinomedical code H32024827, specifications: 2 mL:20 mg) was injected into the areola area. After 5min of pressure, a 3cm-incision was made at the axillary hair stop. The flap is carefully separated, and the blue colored sentinel lymph node was found from the parallel muscle bundle of the lateral pectoralis major muscle and resected. When performing axillary lymph node dissection, operator pay attention to the specific conditions around axillary vascular nerves, fully separate the fascia tissue of the anterior serrate muscle, subscapular muscle and latissimus dorsi, remove the fat layer from the upper axillary vascular sheath to the top of the lower axilla, and from the back to the subscapular muscle and the front of the latissimus dorsi muscle to the back to the pectoralis minor muscle. All the lymph and fat tissue in the axilla are completely removed. The electrocautery group used electrocautery (Zhejiang Shuyou Instrument and Equipment Co., LTD., model: SY-IIIA(N)-1) to perform axillary lymph node surgery, with the same scope and method as the UHS group.

After the wound was rinsed, holes were poked at the anterior axillary line and the midclavicular line. Two silicone drainage tubes were placed in the axilla and breast, respectively. The drainage tubes were connected with negative pressure drainage balls, and pressure bandaging was performed with pressure bandage vests.

Postoperative follow-up was conducted on the occurrence of recent complications of the patients. The follow-up time was limited to 1 month after surgery, during the period of admission and return visit of the patients after surgery.

### Evaluation indicators

Primary outcome: postoperative axillary drainage flow, postoperative axillary drainage tube indwelling time;

Secondary outcome: lymph node operation time, intraoperative blood loss, postoperative pain score [10], and recent postoperative complications.

Among them, the operation time of axilla was recorded as lymph node operation time. The amount of intraoperative blood loss was collected and recorded with aspirator and graduated bottle. The postoperative axillary drainage volume was collected and recorded with negative pressure drainage bottle. When the drainage rate is less than 10ml within 24 hours, the drainage tube can be removed. The pain score on the day after surgery was generally evaluated by digital rating scale (NRS )[11–13]. Recent complications included hematoma, wound infection, delayed wound healing, postextubation effusion and flap necrosis.

### Follow-up Visit

After the operation, the patient was instructed to carry out rehabilitation training as soon as possible. If the drainage tube was not removed when the patient was discharged, the patient was given a record card to record the drainage flow at the designated time at home every day and the occurrence of complications. The patient should place the drainage bag perpendicular to the ground and measure the drainage flow after the liquid level was at a horizontal position. If the drainage bag is full, the drainage tube is leaky or damaged, or the patient has recent complications after surgery, including hematoma, wound infection, delayed wound healing and flap necrosis, they are asked to contact the doctor in time and return to the hospital for appropriate treatment by the designated doctor.

### Statistical Processing

Data of patients were collected. Data were organized and analyzed using SPSS 26.0 statistical analysis software. Normality test was conducted on the data. According to the data type, the mean and standard deviation ($\overline{x}$ ± s) of the numerical variables conforming to normal distribution were described by t test. The numerical variables that did not conform to the normal distribution were described by the median and interquartile distance (M (P25, P75)), and the rank sum test was used. The disordered categorical variables were expressed as percentage (%) and χ2 test was used. Ordered categorical variables are represented by numerical rank, using rank sum test. Sentinel lymph node biopsy, lymph node dissection at stations I,II, and lymph node dissection at stations I, II,III were statistically analyzed and compared among groups. Multiple linear regression analysis is performed between the dependent variable with linear relationship and two or more independent variables. P< 0.05 was statistically significant.

## Results

### Baseline Data

A total of 128 patients were included in this study. The mean age of patients in the UHS group (harmonic group) was 58.28 ± 11.67 years, and that in the electrocautery group was 56.66 ± 11.50 years. The UHS group included 32 patients (50.0%) with TNM stage I, 22 patients (34.4%) with TNM stage II, and 10 patients (15.6%) with TNM stage III. The electrosurgical group included 28 patients (43.8%) with TNM stage I, 25 patients (39.1%) with TNM stage II, and 11 patients (17.2%) with TNM stage III. The mean BMI of patients in the UHS group was 25.63 ± 3.61, and the mean BMI of patients in the electrocautery group was 24.36 ± 3.68. As shown in Table 1, the socio-demographic data of the two groups were comparable (*p*>0.05).

**Table 1:** Baseline Data of the harmonic group and electrocautery group

Characteristics	Harmonic	Electrocautery	*P* value
n	64	64	
Age, mean ± sd	58.28 ± 11.67	56.66 ± 11.50	0.429
AJCC tumor staging, n (%)			0.279
IA	32 (50.0%)	25 (39.1%)	
IB	0 (0%)	3 (4.7%)	
IIA	10 (15.6%)	17 (26.6%)	
IIB	12 (18.8%)	8 (12.5%)	
IIIA	4 (6.3%)	5 (7.8%)	
IIIC	6 (9.4%)	6 (9.4%)	
Clinical TNM stage, n (%)			0.777
I	32 (50.0%)	28 (43.8%)	
II	22 (34.4%)	25 (39.1%)	
III	10 (15.6%)	11 (17.2%)	
BMI, mean ± sd	25.63 ± 3.61	24.36 ± 3.68	0.051
Surgery options, n (%)			0.149
Modified radical mastectomy	52 (81.3%)	45 (70.3%)	
Breast sparing mastectomy	12 (18.8%)	19 (29.7%)	
Comorbidities, n (%)			0.674
DM	5 (7.8%)	5 (7.8%)	
HTN	12 (18.8%)	7 (10.9%)	
CLD	2 (3.1%)	2 (3.1%)	
Histology, n (%)			0.712
Ductal carcinoma	61 (95.3%)	60 (93.8%)	
Lobular carcinoma	2 (3.1%)	1 (1.6%)	
Other	1 (1.6%)	3 (4.7%)	
Family history, n (%)	1 (1.6%)	6 (9.4%)	0.115
Parity	63 (98.4%)	64 (100%)	>0.05
Neoadjuvant therapy, n (%)	4 (6.3%)	8 (12.5%)	0.225
Smoking, n (%)	1 (1.6%)	5 (7.8%)	0.208
Drinking, n (%)	3 (4.7%)	1 (1.6%)	0.619
Extent of lymph node surgery			0.187
SNB	38 (59.4%)	34 (53.1%)	
I,II	15 (23.4%)	21 (32.8%)	
I,II,III	11 (17.2%)	9 (14.1%)	

### The therapeutic effect of UHS group was basically the same as that of electrocautery group

Statistical analysis of all data was performed between the UHS group and the electrocautery group. It showed that there were no significant differences in axillary lymph node operation time, intraoperative blood loss, postoperative axillary drainage volume, axillary drainage tube indwelling time, pain score, and recent postoperative complications (*p*>0.05) . As shown in Table 2.

**Table 2:** Comparison of outcome variables between harmonic group and electrocautery group

Variable	Harmonic (n=64)	Electrocautery (n=64)	*P* value
Duration of axillary surgery (minutes)	13.31 ± 11.86	13.13 ± 10.23	0.924
Blood loss (ml)	21.02 ± 7.51	19.03 ± 3.74	0.062
Total axillary drain volume (ml)	471.47 ± 409.68	487.73 ± 411.38	0.823
Duration of axillary drains (days)	11.73 ± 4.76	11.31 ± 5.67	0.650
Pain score			0.076
1	0 (0%)	1 (1.6%)	
2	35 (54.7%)	24 (37.5%)	
3	29 (45.3%)	39 (60.9%)	
Complications			0.144
Delayed Healing	1 (1.6%)	5 (7.8%)	
Hematoma	2 (3.1%)	3 (4.7%)	
Wound infection	2 (3.1%)	3 (4.7%)	
Flap necrosis	1 (1.6%)	0 (0%)	
Re-accumulation of seroma after drain removal	4 (6.3%)	0 (0%)	

Factors that may affect axillary drainage volume were included in multiple linear regression analysis. Axillary drainage volume was taken as the dependent variable, and other variables were taken as independent variables for multiple linear stepwise regression analysis. Considering the possible interaction between independent variables, interaction terms of independent variables were included in the analysis, α_input_ = 0.05, α_output_ = 0.10. The results are shown in Table 3. Lymph node operation scope and BMI are positively correlated with axillary drainage volume, and the lymph node operation scope has the greatest impact on axillary drainage volume. The other independent variables have no significant impact, and the interaction terms among independent variables are not included in the model. Model test F = 8.327, P < 0.001, coefficient of determination R = 0.409, this model can explain 40.9% of axillary drainage variation.

**Table 3:** Results of multiple linear regression analysis of influencing factors of axillary drainage volume

Variable	B	SEx	Beta	t	p
Age	3.278	2.860	0.093	1.146	0.254
Surgery options	-96.457	74.942	-0.101	-1.287	0.201
Extent of lymph node surgery	175.335	26.449	0.510	6.629	<0.001
Harmonic group and electrocautery group	62.509	59.375	0.077	1.053	0.295
BMI	36.818	7.916	0.332	4.651	<0.001
Comorbidities	-50.946	33.470	-0.107	-1.522	0.131
Histology	-70.705	75.745	-0.065	-0.933	0.353
Family history	138.331	132.917	0.077	1.041	0.300
Parity	259.184	381.912	0.056	0.679	0.499
Neoadjuvant therapy	-83.651	113.940	-0.060	-0.734	0.464
Smoking	135.440	142.665	0.070	0.949	0.344
Drinking	-57.373	194.314	-0.025	-0.295	0.768

Based on the above results, the group was divided into sentinel lymph node biopsy (SNB) group, lymph node dissection group at stations I and II, and lymph node dissection group at stations I,II,III according to the standard of lymph node surgical scope. The statistical analysis results are shown in Tables 4, 5, and 6.

**Table 4:** Comparison of outcome variables between harmonic group and electrocautery group in SNB

Variable	Harmonic (*n*=38)	Electrocautery (*n*=34)	*P* value
Blood loss (ml)	19.34 ± 6.38	19.06 ± 4.48	0.830
Duration of axillary surgery (minutes)	4.79 ± 4.06	4.88 ± 2.85	0.912
Duration of axillary drains (days)	10.53 ± 4.68	8.85 ± 4.56	0.130
Total axillary drain volume (ml)	288.55 ± 169.70	242.47 ± 188.20	0.278
Pain score			0.123
1	0 (0%)	1 (2.9%)	
2	21 (55.3%)	12 (35.3%)	
3	17 (44.7%)	21 (61.8%)	
Complications			0.697
Delayed Healing	1 (2.6%)	3 (8.8%)	
Hematoma	1 (2.6%)	1 (2.9%)	
Wound infection	1 (2.6%)	0 (0%)	
Flap necrosis	0 (0%)	0 (0%)	
Re-accumulation of seroma after drain removal	1 (2.6%)	0 (0%)

**Table 5:** Comparison of outcome variables between harmonic group and electrocautery group in axillary lymph node dissection at station I,II

Variable	Harmonic (*n*=15)	Electrocautery (*n*=21)	*P* value
Blood loss (ml)	21.33 ± 3.51	20.81 ± 3.12	0.643
Duration of axillary surgery (minutes)	24.93 ± 6.87	20.81 ± 6.99	0.088
Duration of axillary drains (days)	13.00 ± 4.78	13.81 ± 6.14	0.673
Total axillary drain volume (ml)	694.60 ± 440.17	741.19 ± 469.05	0.717
Pain score			0.741
1	0 (0%)	0 (0%)	
2	6 (40.0%)	10 (47.6%)	
3	9 (60.0%)	11 (52.4%)	
Complications			0.197
Delayed Healing	0 (%)	2 (9.5%)	
Hematoma	1 (6.7%)	2 (9.5%)	
Wound infection	0 (0%)	3 (14.3%)	
Flap necrosis	0 (0%)	0 (0%)	
Re-accumulation of seroma after drain removal	2 (13.3%)	0 (0%)

**Table 6:** Comparison of outcome variables between harmonic group and electrocautery group at station I,II,III

Variable	Harmonic (*n*=11)	Electrocautery (*n*=9)	*P* value
Blood loss (ml)	26.36 ± 12.06	19.44 ± 1.66	0.088
Duration of axillary surgery (minutes)	26.91 ± 8.83	26.33 ± 5.38	0.860
Duration of axillary drains (days)	14.18 ± 3.89	14.78 ± 4.17	0.745
Total axillary drain volume (ml)	799.09 ± 599.73	799.56 ± 298.95	0.998
Pain score			0.070
1	0 (0%)	0 (0%)	
2	8 (72.7%)	2 (22.2%)	
3	3 (27.3%)	7 (77.8%)	
Complications			>0.05
Delayed Healing	0 (0%)	0 (0%)	
Hematoma	0 (0%)	0 (0%)	
Wound infection	1 (9.1%)	0 (0%)	
Flap necrosis	1 (9.1%)	0 (0%)	
Re-accumulation of seroma after drain removal	1 (9.1%)	0 (0%)	

## Discussion

The SINODAR-ONE randomized clinical trial indicated that in patients with T1-2 breast cancer treated with mastectomy and with one or two giant metastatic sentinel lymph nodes, overall survival and relapse-free survival in patients treated with only SNB were no worse than those treated with ALND. Therefore, in our study, the standard use of SNB for axillary lymph node therapy in these patients is feasible [14]. A retrospective analysis of 291 breast cancer patients undergoing Sentinel lymph node biopsy (SNB) and neo-adjuvant chemotherapy (NAC) was performed. It found that for cN0 and cN+ patients, SLNB was an acceptable procedure in the context of NAC, with an overall good prognosis and a low axillary surgical failure rate [15].

Previous studies have found that the effectiveness of ultrasounic-harmonic scalpel and electrocautery in different types of solid tumors is relatively different, and the advantages and disadvantages of the two devices in terms of performance and safety are still controversial. Studies have shown that the use of UHS during modified radical mastectomy in obese patients can reduce the total drainage flow and reduce the risk of subcutaneous hematoma. But the use of UHS will make the operation time longer [16]. Some researchers believe that compared with electrocautery UHS has certain advantages in reducing blood loss, postoperative drainage volume and drainage days during modified radical mastectomy, and can discharge patients earlier without major complication s[17–20]. However, some studies have found that although the use of UHS in modified radical mastectomy can reduce intraoperative blood loss, total drainage volume, postoperative drainage time and postoperative incidence of subcutaneous hematoma, the average operation time can be significantly extende d[21, 22].

In addition, a retrospective analysis of breast cancer surgery at a regional center showed that the median operation time was shorter in the UHS group. But there were no significant differences between the two groups in terms of length of hospital stay, total wound drainage, total drainage days, and incidence of complications [23]. In a retrospective analysis of modified radical mastectomy in women with locally advanced breast cancer, it was found that the use of Harmonic ultrasounic scalpel and Thunderbeat ultrasounic scalpel did not show significant differences in intraoperative blood loss, subcutaneous hematoma formation, and drainage flow compared with electrocautery [24]. Other studies held similar views, suggesting that lymph vessels should be ligation during axillary lymph node dissection in modified radical mastectomy. UHS could not effectively reduce operation time, intraoperative blood loss, axillary drainage volume and drainage time [25]. Another retrospective analysis conducted at Pusan National University Hospital found that there are no significant difference in intraoperative blood loss and complication rate between the UHS group and electrocautery group [26].

Working principle of two kinds of surgical instruments is studied. The electrocautery generates unipolar electric energy to heat the local tissue through the high-frequency current, and the body tissue is separated, cut and coagulated. The hemostatic effect is good for small blood vessel bleeding, but the hemostatic effect is not good for blood vessels with a diameter of > 2 mm [27]. By converting electrical energy into mechanical energy, the UHS makes the tissue rub at high speed, the water molecules in the tissue vaporize, the tissue breaks off, the protein denatures and coagulates, and the blood vessels close. Approved by the U.S. Food and Drug Administration (FDA), the UHS can safely close blood vessels < 5 mm in diameter. However, some scholars still believe that ligation should be used at the same time to make the hemostatic effect more reliable [28]. In normal adults, the capillary arterial perfusion pressure is about 42cmH_2_O, the capillary venous pressure is about 24cmH_2_O, and the lymphatic pressure is about 7cmH_2_O. Due to the thin wall of the lymphatic tube and the low protein content in the lymph fluid, the coagulation effect of energy instruments is not good. Some scholars suggested that the lymphatic adipose tissue should be ligated and sutured to avoid chylous fistula and lymphatic fistula [29].

In previous studies, the coagulation effect of electrocautery and UHS on lymphatic vessels and blood vessels has been controversial, and most of the previous studies were retrospective studies conducted during modified radical mastectomy. Therefore, our study conducted a prospective study, including unilateral modified radical mastectomy and unilateral breast sparing mastectomy, two of the most widely used surgical procedures in clinical practice. The surgery was performed by the same experienced surgeon to avoid measurement bias. In this study, intraoperative blood loss and operative time of axillary lymph nodes were used to evaluate the coagulation effect of two energy instruments on lymphatic vessels and blood vessels. As the postoperative incision is closed, the coagulation effect of the two surgical energy instruments cannot be directly evaluated. Since the drainage fluid on the first day after surgery contains blood contents and high concentration of creatine phosphokinase, and then mostly lymph fluid [30], the postoperative axillary drainage volume, the postoperative retention time of axillary drainage tube, the postoperative pain score and the occurrence of recent postoperative complications were used to indirectly evaluate the effect of two kinds of surgical energy instruments on the coagulation of lymphatic vessels and blood vessels after operation.

The results of our study showed that compared with the electrocautery group, there were no significant differences in axillary lymph node operation time, intraoperative blood loss, axillary drainage catheter indwelling time after operation, axillary drainage volume, pain score, and recent postoperative complications in UHS group(p>0.05). In this study, factors that may affect axillary drainage volume were included in multiple linear regression analysis, which showed the influence of a factor on axillary drainage volume after controlling other factors, and quantified the relationship between influencing factors and axillary drainage volume [31]. The results showed that lymph node operation scope and BMI were positively correlated with axillary drainage volume (p<0.001), and the extent of lymph node operation had the greatest influence on axillary drainage. Therefore, in order to further clarify whether there were differences between and within groups, our study conducted a grouping study based on the different scopes of lymph node surgery. Patients were divided into sentinel lymph node biopsy (SNB) group, lymph node dissection group at stations I and II, and lymph node dissection group at stations I,II,III according to the area of axillary lymph node operation. The differences between the UHS group and the electrocautery group were compared in each group. The results showed that in each group, there were no significant differences in axillary lymph node operation time, intraoperative blood loss, axillary drainage tube indwelling time, axillary drainage volume, pain score, and recent postoperative complications between the UHS group and electrocautery group (p>0.05). When the same surgeon used UHS and electrocautery respectively for axillary lymph node surgery, there was no significant difference in the immediate hemostatic effect of the two surgical energy instruments, and the cutting efficiency was roughly the same. The use of two different energy instruments did not significantly affect the duration of surgery. The axillary drainage volume was significantly affected by the lymph node operation scope and BMI, but was less affected by the choice of surgical method and surgical energy instruments. In addition, there was no significant difference in postoperative retention time of axillary drainage tube, postoperative incision pain perception, and the probability of near-term postoperative complications were roughly the same between the two groups. Studies have shown that axillary drainage flow and axillary drainage tube indwelling time are closely related to postoperative lymphedema occurrence, postoperative recovery time, and tumor prognosis of patient s[32–34].

At present, electrocautery and UHS are the most widely used surgical instruments in various clinical departments. But there is still a large gap in the guidelines for the use of surgical energy instruments such as breast cancer and gastric cancer. Our study can provide certain references for subsequent clinical research and the selection of clinical instruments. The cost of a single use of the UHS is about 2000 yuan (about $278). The price of a single use of the electrocautery is about 100 ~ 200 yuan (about $13~27), which is much lower than that of the UHS. According to the experimental results of our study, it can guide clinicians to rationally select surgical energy instruments to perform the operation, which can reduce the cost consumption and save medical resources and reduce the burden of patients' families and social economy., while promoting the good recovery of patients after the operation.

## Conclusion and prospect

In summary, there is no significant difference between the efficacy of UHS and electrocautery in axillary lymph node treatment for breast cancer patients undergoing radical surgery. For breast cancer patients who treated with SNB, surgeons are recommended to choose to use electrocautery for unilateral modified radical mastectomy and unilateral breast sparing mastectomy. For breast cancer patients who need treatment with ALND, electrocautery is also recommended. Unless it does not close the blood vessel well. The advantage is that, without compromising the effectiveness of the surgery, electrocautery can reduce the prolongation of operation time caused by intraoperative instrument replacement and the probability of other intraoperative accidents, while reducing the cost of surgery.

There are some shortcomings in this study: this study is a single-center prospective study with relatively limited sample sources, which cannot avoid the conclusion bias caused by factors such as geographical location and choice of medical places for patients. Another disadvantage is that the follow-up time is short. Only the occurrence of recent postoperative complications is counted. The tumor prognosis of the two groups cannot be predicted and compared. Randomized NSABP B-32 trials reported significant differences in a range of upper limb morbidity, including shoulder abduction, numbness, and arm tingling [35]. But data on how SLB compares to no axillary surgery is still scarce. The randomized INSEMA trial showed that patients in the group that did not have any axillary surgery experienced fewer arm symptoms, which was a statistically significant difference [36]. In the SOUND trial, one week after surgery, arm and shoulder symptoms increased significantly faster in the SNB group than in the observation-only group [37]. Definitive results over a longer follow-up period need to be discovered. Future multi-center, long-term studies with more patients and longer follow-up periods can be conducted to verify whether the conclusions of this study are generalizable.

## Data Availability

The datasets used and/or analyzed during the current study are available from the Department of General Surgery, Zhongda Hospital Affiliated to Southeast University, on reasonable request.
